# Statistical risk prediction models for adverse maternal and neonatal outcomes in severe preeclampsia in a low-resource setting: proposal for a single-centre cross-sectional study at Mpilo Central Hospital, Bulawayo, Zimbabwe

**DOI:** 10.1186/s13104-019-4539-y

**Published:** 2019-08-13

**Authors:** Solwayo Ngwenya, Brian Jones, Alexander Edward Patrick Heazell, Desmond Mwembe

**Affiliations:** 1 Department of Obstetrics & Gynaecology, Mpilo Central Hospital, P.O. Box 2096, Vera Road, Mzilikazi, Bulawayo, Matabeleland Zimbabwe; 2 Royal Women’s Clinic, 52A Cecil Avenue, Hillside, Bulawayo, Zimbabwe; 3 National University of Science and Technology, Medical School, P. O. Box AC 939, Ascot, Bulawayo, Matabeleland Zimbabwe; 4 Tommy’s Research Centre, Manchester Academic Health Science Centre, Faculty of Biology, Medicine and Health, The University of Manchester, St Mary’s Hospital, Oxford Road, 5th Floor (Research), Manchester, M13 9WL UK

**Keywords:** Preeclampsia, Eclampsia, Maternal mortality, Perinatal mortality, Maternal morbidity, Neonatal mortality, Prediction

## Abstract

Hypertensive disorders in pregnancy are a leading cause of maternal and perinatal morbidity and mortality, especially in low-resource settings. Identifying mothers and babies at greatest risk of complications would enable intervention to be targeted to those most likely to benefit from them. However, current risk prediction models have a wide range of sensitivity (42–81%) and specificity (87–92%) indicating that improvements are needed. Furthermore, no predictive models have been developed or evaluated in Zimbabwe. This proposal describes a single centre retrospective cross-sectional study which will address the need to further develop and test statistical risk prediction models for adverse maternal and neonatal outcomes in low-resource settings; this will be the first such research to be carried out in Zimbabwe. Data will be collected on maternal demographics characteristics, outcome of prior pregnancies, past medical history, symptoms and signs on admission, results of biochemical and haematological investigations. Adverse outcome will be defined as a composite of maternal morbidity and mortality and perinatal morbidity and mortality. Association between variables and outcomes will be explored using multivariable logistic regression. Critically, new risk prediction models introduced for our clinical setting may reduce avoidable maternal and neonatal morbidity and mortality at local, national, regional and international level.

## Introduction

Preeclampsia occurs only in human pregnancy and is characterised by high blood pressure and significant proteinuria after 20 weeks’ gestation [1]. There is a general agreement to define preeclampsia as severe if blood pressure was ≥ 160 mmHg systolic or 110 mmHg diastolic [2–4]. von Dadelszen et al. defined preeclampsia as occurring after 20 weeks’ gestation with high blood pressure (i.e. BP > 160–170/100–110), significant proteinuria of > 3–5 g/24 h, and/or the occurrence of symptomatology, such as headache or visual disturbances [5]. Severe preeclampsia causes significant adverse impact on maternal, fetal and neonatal health. Critically, avoidable maternal and neonatal morbidity and mortality may result particularly in cases of severe disease.

It is estimated that 50,000–100,000 annual maternal deaths attributable to these conditions globally, as well as 500,000 fetal and neonatal deaths [6], including increased risks of fetal growth restriction and stillbirth [7].

According to Say et al. the three most common causes of maternal deaths globally as of 2010 are haemorrhage, hypertensive disorders and sepsis, accounting for more than half of maternal deaths [8]. In 2015 developing countries accounted for approximately 99% (302,000) of the global maternal deaths, with sub-Saharan Africa alone accounting for roughly 66% (201,000) as reported by the WHO (World Health Organisation) et al. [9]. The same WHO report states that critically most of the deaths were avoidable if they had care and access to healthcare.

In Zimbabwe, hypertensive disorders were the third leading cause of maternal deaths [10]. The overall incidence of severe preeclampsia and eclampsia at Mpilo Central Hospital in 2017 was 1.3% [11]. The incidence of severe preeclampsia has been reported to be 0.38% in the United States of America, with chronic hypertension and congenital anomalies strongly associated with preeclampsia as found by Lisonkova et al. and 13% by Pettit et al. [12, 13]. Abalos et al. found that the overall incidence of preeclampsia in Brazil was 1.5% [14].

Iacobelli et al. and Robillard et al. reported that the predominance of early-or late onset preeclampsia has huge geographical differences [15, 16]. Ratsiatosika et al. in a study in Madagascar found a high overall incidence of early-onset preeclampsia of 37% versus approximately 10% in the international literature [17]. The study also found high rates of early-onset preeclampsia in Guadeloupe (31%), Reunion (31%), Mauritius (34%), Cameroon (37.4%), China (38%), Zimbabwe (58%), Thailand (34%), Turkey (29%) and India (26%). Sansone et al. found that human immunodeficiency virus (HIV)-infected women were at an increased risk of preeclampsia [18].

Despite all the research published in the last three decades on screening and prevention of preeclampsia, the condition remains one of the main causes of maternal and perinatal morbidity and mortality, both in low and high-income countries. Rolnik et al. reported that preeclampsia affects 2–8% of pregnancies [19]. Dekker and Sibai noted that proper antenatal care and timed delivery are of utmost importance in tertiary prevention of preeclampsia [20]. The Collaborative Low-dose Aspirin Study in Pregnancy (CLASP) suggested that aspirin could be effective in reducing the risk of recurrent early-onset preeclampsia, if started before 32 weeks gestation as reported by de Swiet [21]. A problem observed in low-income settings is that women with identifiable risk factors for developing hypertensive disorders of pregnancy cared for in inappropriate city health clinics or rural areas. Consequently, they do not receive antenatal therapy such aspirin therapy where this is clearly indicated or have regular surveillance of their blood pressure or proteinuria. They are usually referred as dire emergencies and this results in poor perinatal outcomes.

Consequently, a better understanding of predictors of severe preeclampsia may improve maternal and neonatal morbidity by facilitating access to preventative measures, focused antenatal care or timely delivery. Against this background, the literature shows that models have been developed to help mitigate the effects of severe preeclampsia on maternal and neonatal health.

## Problem statement

Severe preeclampsia has very poor outcomes for women and neonates in low-resource settings. Consequently, hypertensive disorders cause a huge burden on the healthcare as they were the third leading cause of maternal deaths in Zimbabwe in 2007 [10]. However, there are a few such predictive models which are applicable to the local population and there are no locally developed or evaluated statistical risk prediction models. There is paucity of data derived from low-resource settings to study this important subject even though the disease mainly adversely affects pregnant women from low-resource settings such as those in Bulawayo. The non-availability of predictive models is one of the precipitating factor in adverse outcome. This inability to predict women whose pregnancy will end in adverse maternal and neonatal outcome, deprives pregnant women and their babies’ potential preventative treatment and management strategies that will improve outcomes.

## Justification of the study

Hypertensive disorders in pregnancy are a leading cause of maternal and perinatal morbidity and mortality especially in low-resource settings. Preeclampsia risk prediction models can help in triaging and managing patients promptly hence potentially saving lives. The best available models such as the fullPIERS model (Preeclampsia Integrated Estimate of RiSk) were developed in high-resource settings and used variables which are unavailable in low-resource countries. Subsequent developments, including miniPIERS used data from low- and middle-income countries (LMICs). However, there are no such risk prediction models that have been developed for our local settings in Bulawayo or Zimbabwe. This study will address the need to develop and test statistical risk prediction models in a relevant local population.

This will be the first time such research to produce risk prediction models would be carried out at a local or national level setting in Zimbabwe. It is anticipated that remote rural areas in our setting could use such a model to predict preeclampsia risks and refer patients early. In women already attending the Central facility, women at greatest risks of complications could be identified and treatment initiated promptly. Implementation of predictive models could then be prospectively evaluated to determine whether this improves outcomes for women and their babies.

For practical reasons the models will be developed using data which are already routinely and easily collected and are available for use. Due to resource constraints, only standard laboratory tests will be included in the models to ensure they are appropriate in our low-resource setting. Therefore, this makes the development of such models achievable. Clinicians will likely find the models useful as the predictor variables are encountered in their daily work.

Crucially, new risk prediction models introduced for our clinical setting may reduce avoidable maternal and neonatal morbidity and mortality at local, national, regional and international level.

## Aim

To develop and validate simple clinical risk prediction models for predicting adverse maternal and neonatal outcomes in severe preeclampsia in a low-resource setting.

### Objectives

- To determine the incidence and associated risk factors of severe preeclampsia in a low-resource setting.
- To investigate the demographic contributions of severe preeclampsia in a low-resource setting to poor maternal and neonatal outcomes.
- To develop statistical risk prediction models for predicting adverse maternal and neonatal outcomes in severe preeclampsia in a low-resource settings.
- To compare and validate the developed maternal model to the miniPIERS.

## Literature review

Schummers et al. reported that, compelled by the intuitive appeal of predicting each individual woman’s risk of an adverse outcome, there is a growing interest in risk prediction models [22]. In a systematic review, Al-Rubaie et al. noted that statistical risk prediction models are valuable in identifying women at risk of preeclampsia to guide management, but that specialized models have significantly better performance than simple ones [23]. Importantly, risk-prediction models have been developed in a limited number of settings and there are no such risk prediction models for preeclampsia developed or validated in our low-resource setting at Mpilo Central Hospital or in Zimbabwe. Models developed elsewhere where resources are rich may not be appropriate for our setting as many patients may come from rural settings or have limited antenatal care. Furthermore, risk prediction models developed in rich-resourced settings also use predictor variables such as laboratory markers which are not routinely done in low-resource settings.

Risk prediction models can use routinely collected maternal characteristics to predict risks. Routinely collected maternal characteristics include maternal age, parity, marital status and history of hypertensive disorders some of which are known to be associated with the development of hypertensive disorders of pregnancy. It is important to note that most of the prediction models for preeclampsia focus on maternal outcomes and with no mention on neonatal outcomes.

Ukah et al. concluded that the ability to predict severe early-onset preeclampsia using simple tests could aid in the management of severe preeclampsia and improve outcomes [24]. In low-resource settings, such risk prediction models could help rural healthcare workers predict disease progression and refer patients earlier rather than later in emergency situations.

von Dadelszen et al. produced the best known model to predict adverse maternal outcomes in hypertensive disorders of pregnancy called the fullPIERS model [25]. It was developed for predicting adverse maternal outcomes from 2023 women with preeclampsia using data from tertiary centres in high-income countries (Canada, New Zealand, Australia and the UK), and used maternal demographics, signs, symptoms and laboratory tests as predictors. It had good discrimination with an area under receiver operating characteristic curve (AUROC) of 0.88, 95% CI 0.84–0.92, sensitivity 76% and specificity 87%. fullPIERS accurately predicted adverse maternal outcomes for up to 48 h, a clinically useful period that allows corticosteroids administration, in utero transfer or induction. It showed both internal and external validities for predicting adverse maternal outcomes within 48 h for women admitted with preeclampsia at any gestational age. Ukah et al. found that the ability to recognize women at highest risk of complications earlier could aid in preventing these adverse outcomes through improved management [26, 27].

The miniPIERS model was developed for low- and middle-income countries using data of 2081 women from Fiji, Uganda, South Africa, Brazil and Pakistan. This logistic regression model was developed to provide a simple, evidence-based tool to identify pregnant women in LMICs at increased risk of death or major hypertensive-related complications. This model included parity, gestational age on admission, headache/visual disturbances, chest pain/dyspnoea, vaginal bleeding with abdominal pain, systolic blood pressure and urine proteinuria [28]. It had good discrimination, albeit lower than the fullPIERS model, with an area under curve of receiver operating characteristic (AUROC) of 0.768, 95% CI 0.735–0.80. However, the sensitivity was much lower at 41.4% and specificity 91.9%. Individual country analysis showed some variation such that South Africa had an AUROC of 0.762, 95% CI 0.702–0.821 and in Uganda the AUROC was 0.656, 95% CI 0.523–0.799.

Thangaratinam et al. did a (prediction of complications in early-onset pre-eclampsia-logistic regression) PREP-L model with data from 946 women from 53 hospitals in England and Wales [29]. The model included: maternal age, gestation, medical history, systolic blood pressure, deep tendon reflexes, urine protein creatinine ratio, platelets, serum alanine amino transaminase and creatinine. The model showed an optimism-adjusted c-statistic of 0.82 (95% CI 0.80 to 0.84) for composite adverse maternal outcomes by 48 h. The model used estimated fetal weight and liquor volume by ultrasound scan, uterine artery Doppler, cardiotogography findings and administration of steroids for prediction of fetal outcome. Thangaratinam et al. noted that high-resource settings studied preeclampsia risk prediction models have a potential role in triaging high risk mothers who may need transfer to tertiary units for intensive maternal and neonatal care [30], which would still be a laudable goal in the Zimbabwean context.

Onwudiwe et al. used multiple regression analysis to demonstrate that various maternal characteristics such as uterine artery Doppler and mean arterial pressure provided significant independent contribution in the prediction of preeclampsia with a false-positive rate of 10%, the estimated detection rates of early- and late-onset preeclampsia were 100% and 56.4% respectively [31]. As stated earlier, Al-Rubaie et al. validated simple preeclampsia risk models and demonstrated good risk discrimination achieving the highest AUROC (0.76, 95% CI 0.74–0.77) [23].

Ukah et al. found that the most promising prediction was with multivariable models [27]. However, von Dadelszen et al. used a multiple logistic regression model that revealed gestational age on admission to hospital (Odds Ratio (OR) OR, 0.91), dipstick proteinuria (OR, 1.31), and mean platelet volume: platelet ratio (OR, 391.0) independently predicted adverse maternal outcomes in preeclampsia [32].

Thangaratinam et al. used logistic regression models to assess the overall risk of any maternal or neonatal outcome and a survival analysis model to obtain individual risk estimates [29]. Other researchers have used statistical models including maternal age, gestation, medical history, systolic blood pressure, deep tendon reflexes, and urine protein to creatinine ratio, platelets, serum alanine amino transaminase, urea, creatinine, oxygen saturation and treatment with antihypertensives or magnesium sulphate. In another example of risk prediction model from a high-resource setting, Gabbay-Benziv et al. found probability scores considering nulliparity, prior preeclampsia, body mass index, diastolic blood pressure and placental growth factor had an AUROC of 0.784 (95% CI 0.721–0.847) [33]. In low-resource settings, due to limited funding in healthcare, some of the biochemical characteristics are not routinely measured hence some cannot be included in the risk prediction models for our locally developed models. Models for low-resource settings e.g. miniPIERS focus on maternal characteristics such as: parity, gestational age on admission, headaches/visual disturbances, chest pain/dyspnea, vaginal bleeding with abdominal pain, systolic blood pressure and urine proteinuria in their model [28].

Almedia et al. validated the fullPIERS and showed an AUROC of 0.72 (*P *< 0.001), determining a cut-off point for fullPIERS probability of 1.7% [34]. In this population, sensitivity of miniPIERS was 60.0% and specificity was 65.1%; the positive likelihood ratio was 1.72 and the negative likelihood ratio was 0.61. The sensitivity implies that 40% of cases of preeclampsia are not predicted at all. The miniPIERS model was well-calibrated and had an AUROC of 0.768 (95% CI 0.735–0.801) with an average optimism of 0.037. Caradeux et al. did a risk prediction model for early-onset preeclampsia with a 5% false positivity and achieving a sensitivity of 62.5% and specificity of 95.5% [35].

The fullPIERS model performed well in the prediction of adverse maternal outcomes in women with preeclampsia but crucially did not attempt to predict neonatal outcome. It is easy to use. The model by Agrawal and Maitra was based on important clinical and biochemical parameters and does not require extensive laboratory testing [36]. This research will develop models for low-resource-settings using patients’ data from Bulawayo to predict risks applicable to patients in a low-resource setting.

This research’s predictor variables will include maternal characteristics, simple bedside and laboratory tests, therapeutic interventions and fetal characteristics similar to the fullPIERS except expensive laboratory tests like detailed renal and liver tests or placental growth factor. It will also be similar to the miniPIERS in terms of low- and middle-income countries settings, but this research will include some basic laboratory tests (haemoglobin, platelets and alanine transaminase) and therapeutic interventions that were not included in the miniPIERS (see Table 1). The model by Thangaratinam et al. was similar in terms of most characteristics but differing in the inclusion of oxygen saturation [29]. Crucially, all these other models only predicted adverse maternal outcome except the one by Thangaratinam et al. This research will predict both adverse maternal and neonatal outcomes in a low-resource setting for the first time using fewer laboratory tests than those done by Thangaratinam et al. due to the difference in the availability of resources [30]. This research will be published as mpiloPIERS, after Mpilo Central Hospital where it is being carried out.

**Table 1:** Examples of predictive models on adverse maternal or neonatal outcomes

Author	Year	Country	Predictor variables	Outcome	AUROC	Sensitivity (%)	Specificity
von Dadelszen et al.	2011	Canada, New Zealand Australia UK	Demographic characteristics Clinical Interventions Pregnancy outcomes	Maternal	0.880	76	87%
Payne et al.	2014	Fiji Uganda South Africa Brazil Pakistan	Demographic characteristics Symptoms Signs	Maternal	0.768	41.4	91.9%
Thangaratinam et al.	2017	England Wales	Demographic characteristics Medical history Signs Laboratory tests Oxygen saturation Antihypertensives Magnesium sulphate	Maternal Neonatal	0.840	82	–

## Methodology design

### Study type, setting and participants

The study will employ a retrospective cross-sectional design and will be carried out at Mpilo Central Hospital, a government teaching and tertiary referral centre. Some of the participants will overlap with published studies on the same subject [11, 37]. Mpilo Central Hospital is located in Bulawayo. Bulawayo, located in Matabeleland is the second largest city in Zimbabwe after the capital city Harare, with a population of 653, 337 as of the 2012 census [38]. Mpilo Central Hospital is a 1000-bedded hospital and its maternity unit delivers 8000–10,000 babies per year. This research proposal is for a PhD research project that is registered with the National University of Science and Technology. It will cover the period from January 1, 2016 to December 31, 2018.

### Inclusion and exclusion criteria

Participants will be included in the study if they have a diagnosis of severe preeclampsia. Severe preeclampsia will be defined as high blood pressure [systolic blood pressure (SBP) ≥ 160, diastolic blood pressure (DBP) ≥ 110 mmHg] and or either severe headaches, epigastric pains and deranged biochemical/haematological blood indices. Both singleton and twin/higher order pregnancies will be included. Women with mild or moderate preeclampsia or less than 20 weeks’ of gestation and those with epilepsy will be excluded from the study.

### Main outcome measure

The outcome of interest for this study will be maternal death or serious morbidity (composite adverse maternal outcome) and perinatal death (stillbirth + early neonatal death (defined as death within 7 days of birth) or serious morbidity (composite adverse neonatal outcome) [37]. Maternal morbidity is defined as one or more serious complication of major organ morbidity in renal, hepatic, cardiac, respiratory, cerebral and haematological systems, pulmonary oedema, ventilator support, renal dialysis, transfusion of any blood product, abruption placenta, antepartum haemorrhage and postpartum haemorrhage within 48 h of admission to 7 days post-delivery [39]. The composite adverse neonatal outcome will be defined as one or more of perinatal mortality, 5 min Apgar score < 7, respiratory distress syndrome and admission to neonatal intensive unit.

### Data collection and tool

Data collection will be initially be achieved using a paper data collection tool (Appendix 1). It will be used to collect secondary data from the labour ward delivery registers, perinatal registers and mortality registers. The data will be collected primarily by the researcher and double entered to prevent errors. Data will also be collected from neonatal intensive care unit and special care baby unit. Hospital case notes will be retrieved and the clinical data collected.

### Study design and initial analysis

#### Sample size

Simple proportion formula will be used to calculate the sample size, with the following assumptions 95% confidence interval (CI) and a margin of error of 5%. In the 5 years (2014–2018) to be studied roughly 40,000 deliveries will be analysed. The overall incidence of severe preeclampsia/eclampsia was 1.3% in the unit [11]. The final sample will be around 500 but may be more as all the available cases during the study period will be included.

#### Variables to be considered for the models

Some of these variables are similar to those considered under the miniPIERS and fullPIERS models. This will allow some comparisons to be made to the models developed from this research (see Table 2).

**Table 2:** Maternal, Pregnancy and Fetal variables to be collected for the predictive model

Characteristic	
Maternal demographic characteristics	Maternal age (years)
	Gravidity
	Parity
	Marital status
	Level of education
	HIV status
	Anti-retroviral status
Pregnancy characteristics	Booking status
	Gestation on admission
	Number of fetuses
Past obstetrics history	Aspirin therapy
	History of previous hypertensive disorder
Past medical history	Pre-existing disease of hypertension
	Pre-existing disease of diabetes mellitus
	Pre-existing renal disease
Area of dwelling	Urban/rural
Symptoms/signs	Nausea/vomiting
	Frontal headaches
	Epigastric pains
	Visual disturbances
	Right upper quadrant pains
	Vaginal bleeding with abdominal pains
	Chest pains
	Convulsions
Cardiovascular signs	Systolic blood pressure on at diagnosis (mmHg)
	Diastolic blood pressure on at diagnosis (mmHg)
Haematological tests	Haemoglobin level (g/dl)
	Platelet count (×10^9^/l)
Renal tests	Urine dipstick proteinuria
Hepatic tests	Alanine transaminase (U/l)
Therapeutic	Antihypertensive therapy
	Magnesium sulphate therapy
	Corticosteroid therapy
Fetal characteristics	Fetal heart rate
	Apgar scores
	Admission to neonatal intensive care unit
	Respiratory distress syndrome

Candidate predictor variables for the final model development will be those variables that will be either of (i) available and easy to collect in our settings including in rural health centres, (ii) those that are known to be associated with preeclampsia and (iii) those that are measurable, simple and reliable methods even in rural health clinics, as in the miniPIERS model by Payne et al. [28].

#### General statistical analysis

The data will be entered into a Microsoft Excel Inc. spreadsheet. Data will be exported to the SPSS Version 20 (IBM Corp., Armonk, NY, USA) for analysis. Univariate statistical analysis will be used and presented as frequencies and percentages for categorical variables. Continuous variables will be checked for normal distribution using the Shapiro–Wilk test and mean and standard deviation (SD) will be reported for all data. For variables not normally distributed, non-parametric tests such as the Wilcoxon tests will be used. Bivariate statistical analysis will be used to test for association between independent and dependent variables, using the Pearson or Spearman two-tailed Chi square tests. This will test any statistical associations between the explanatory variables with the composite maternal and neonatal outcomes. A *P* value of < 0.05 would be considered statistically significant.

### Risk prediction regression model development

#### Predictor variables

Predictor variables will include the maternal characteristics, simple bedside and laboratory tests, therapeutic interventions and fetal characteristics outlined in “Variables to be considered for the models” above. Continuous variables like maternal age will be put in groups for analysis before logistic regression. Multiple imputation will be used for missing data. Multiple imputation will allow for the uncertainty about missing data, a process found in SPSS Version 20 package.

#### Composite adverse maternal and neonatal outcomes

The composite adverse maternal outcome to be predicted by the model will be determined by the Delphi consensus as described by Brown et al. and will include maternal mortality or one or more serious complication of major organ morbidity in renal, hepatic, cardiac, respiratory, cerebral and haematological systems, renal dialysis, transfusion of any blood product, abruption placenta, antepartum haemorrhage and postpartum haemorrhage within 48 h of admission to 7 days post-delivery [39]. The composite adverse neonatal outcome will be determined by the Delphi consensus and defined as one or more of perinatal mortality, 5 min Apgar score < 7, respiratory distress syndrome and admission to neonatal intensive unit. The relationship between each predictor variable and the composite adverse maternal or neonatal outcome will first be assessed by binary logistic regression. The Hosmer–Lemeshow goodness-of- fit for logistic regression models with be used. Backward elimination regression models will be used to build models with a stopping rule of *P *< 0.20. Predictor variables with a *P* value of < 0.2 will be considered for the final binary logistic regression models. Binary logistic regression models will be used to predict the adverse maternal outcome or neonatal outcome.

#### The final models

In developing the final binary logistic regression models (logit), the predictor variables with a *P* value of < 0.2 will be considered for the following models;

$${\text{logit y}} = e^{{\beta_{0} + \mathop \sum \limits_{i = 1}^{k} \beta_{i } x_{i} }}$$

where y is the binary dependent variable (adverse maternal outcome or neonatal outcome), $\beta_{0}$ is the constant when all variables are equated to zero, $\beta_{i}$ is the ith coefficient for variable i, i = 1, 2, 3…, k. $x_{i}$ is the ith independent variable.

#### Assessment of model’s performance and validation

Calibration ability of the model will be assessed visually by plotting deciles of predicted probability of an adverse maternal outcome against the observed rate in each decile and fitting a smooth line as done by Harrell et al. and Steyerberg et al. [40, 41]. Performance of the models will be assessed using the area under the curve (AUC) of the receiver operating characteristic (ROC). Standard bootstrapping techniques will be used to assess potential over-fitting. Discrimination ability will be evaluated on the basis of area under curve of the receiver operating characteristic (AUROC) as stated by Hanley and McNeil [42]. Internal validation of the model will be assessed using Efron’s enhanced bootstrap method described by Efron and Tibsherani [43]. External validation will be assessed using the miniPIERS model.

## Data Availability

Not applicable.

## Abbreviations

CLASP: Collaborative Low-dose Aspirin Study in Pregnancy
fullPIERS: full Preeclampsia Integrated Estimate of RiSk
HIV: human immunodeficiency virus
LMICs: low-middle income countries
minillPIERS: mini Preeclampsia Integrated Estimate of RiSk
OR: odds ratio
PREP-L: prediction of complications in early-onset pre-eclampsia-logistic regression
WHO: World Health Organisation
